# Molecular Detection of *Pythium insidiosum* in Cutaneous Lesions of Horses from Northeastern Brazil

**DOI:** 10.3390/ani15192863

**Published:** 2025-09-30

**Authors:** Artur Bibiano de Vasconcelos, Danilo Alves de França, Ana Carolina do Prado, Danielle Hamae Yamauchi, Andrezza Caroline Aragão da Silva, Isabella de Oliveira Barros, Sandra Regina Fonseca de Araújo Valença, Simone Baldini Lucheis, Sandra de Moraes Gimenes Bosco

**Affiliations:** 1 Department of Animal Production and Preventive Veterinary Medicine, School of Veterinary Medicine and Animal Science, São Paulo State University, Valter Maurício Corrêa, Botucatu 18618-681, Brazil; ana.prado@unesp.br (A.C.d.P.); danihyamauchi@gmail.com (D.H.Y.); simone.b.lucheis@unesp.br (S.B.L.); sandrabosco159@gmail.com (S.d.M.G.B.); 2 Department of Preventive Veterinary Medicine and Animal Health, School of Veterinary Medicine and Animal Science, University of São Paulo, Orlando Marques de Paiva, São Paulo 05508-270, Brazil; danilo.af@usp.br; 3 Department of Parasitology and Microbiology, School of Veterinary Medicine, Federal University of Piauí, Avenida Universitária, Teresina 64049-550, Brazil; andrezzaaragaovet@hotmail.com; 4 Department of Microbiology, Centre for Biotechnology, Federal University of Paraíba, Cidade Universitária, João Pessoa 58051-900, Brazil; doutorabella@hotmail.com; 5 Department of Biology, School of Veterinary Medicine, Federal Rural University of Pernambuco, Dom Manoel de Medeiros, Recife 52171-900, Brazil; sandra.araujo@ufrpe.br

**Keywords:** equine, granulomatous lesions, nested-PCR, pythiosis

## Abstract

Pythiosis is an underestimated and neglected disease in Brazil, both in horse breeders and in horses. The molecular detection of *P. insidiosum* in horses in the Brazilian Northeast represents a milestone in the epidemiology of equine pythiosis in the country. This study reports novel cases of equine pythiosis, diagnosed by molecular methods, in five states of Northeastern Brazil. Clinical samples were submitted to microbiological culture, DNA extraction, and nested-PCR for molecular detection of *P. insidiosum*. The nested-PCR successfully detected *P. insidiosum* in four out of five equine lesion samples, demonstrating higher sensitivity compared to microbiological culture, which confirmed the pathogen in only one case. These findings reinforce the effectiveness of molecular diagnosis for equine pythiosis compared to conventional methods. This is the first molecular confirmation of *P. insidiosum* in horses from Northeastern Brazil, emphasizing the need for broader surveillance and improved diagnostic approaches in neglected regions.

## Simple Summary

Pythiosis is a serious but often overlooked disease that affects horses living in flooded or swampy areas. It is caused by a microorganism found in water, which infects the skin and produces large, painful wounds that are difficult to heal and can be fatal without proper treatment. In this study, we describe cases of horses from Northeastern Brazil with skin lesions suspected of pythiosis. Using a modern DNA-based test, we confirmed the infection in four animals. These are the first molecularly confirmed cases in this region, highlighting the need for faster and more reliable diagnostic tools to improve horse health and welfare.

## 1. Introduction

Pythiosis is a chronic pyogranulomatous disease affecting various animal species and humans, caused by the oomycete *P. insidiosum* [1]. This microorganism thrives in aquatic environments like swamps and flooded fields [2]. Zoospores, upon contact with animal skin, encyst and form germ tubes, initiating hyphae development that causes tissue destruction and the formation of masses known as kunkers. In chronic cases, *P. insidiosum* hyphae are typically restricted to these kunkers [3].

Thailand has the highest prevalence of human pythiosis, and Brazil has the highest prevalence of equine pythiosis [4,5,6]. Research on equine pythiosis in Brazil is limited, with few serological and molecular studies. A recent systematic review compiling six decades of reports highlighted that most available data originate from the South and Central regions, with scarce information from the North and Northeast regions, despite these areas presenting favorable environmental conditions for disease occurrence. The review also showed that cutaneous lesions are the most frequently reported clinical form in horses, and that diagnosis is still largely based on clinical and histopathological findings, with molecular confirmation remaining restricted [7].

Recent studies have provided additional insights into the epidemiology and diagnosis of equine pythiosis in Latin America. In the Brazilian Pantanal, a seroepidemiological survey identified a prevalence of 12.5% in horses and highlighted the complex relationship between the host, the pathogen, and aquatic environments that favor disease transmission [8]. More recently, epidemiological surveys in the South of the country indicated a prevalence of 11% [9], and molecular studies confirmed the pathogen in skin lesions of horses by nested-PCR, which is considered more sensitive and specific than isolation [10].

Beyond Brazil, the first molecularly confirmed case of equine pythiosis was recently reported in Northern Veracruz, Mexico, expanding the known geographic distribution of the disease in the Americas and reinforcing the role of molecular tools in confirming clinical suspicion [11]. Together, these findings underscore the emergence of equine pythiosis as a relevant health problem for horses in tropical and subtropical regions and highlight the importance of combining epidemiological data with molecular confirmation.

In Northeastern Brazil, the diagnosis of equine pythiosis has relied mainly on clinical evaluation and histopathology, which may lead to uncertainties in the differential diagnosis with conditions such as habronemosis, zygomycosis, and equine sarcoid. This is illustrated in the study by Souto et al. [12], which reported 1331 suspected cases of equine pythiosis between 1985 and 2020, of which 202 were confirmed through microbiological and histochemical methods [12,13,14]. Molecular studies are needed to clarify the presence of *P. insidiosum* in lesions and enhance understanding of the disease’s epidemiology in this region. This study aimed to report the detection of *P. insidiosum* in clinical skin samples and kunkers from horses in Northeastern Brazil using nested-PCR.

## 2. Materials and Methods

Five clinical samples of equine lesions suggestive of pythiosis were acquired from horses from five states in Northeastern Brazil. Each tissue sample was collected from a different animal that exhibited a characteristic granulomatous skin lesion. The samples were placed in a 50 mL universal collecting flask containing saline 0.9% (Sigma-Aldrich, St. Louis, MO, USA) and chloramphenicol 50 μg/mL (Sigma-Aldrich, St. Louis, MO, USA), and processed. The origin of the samples, lesion location and animal data can be seen in Table 1.

**Figure 1:**
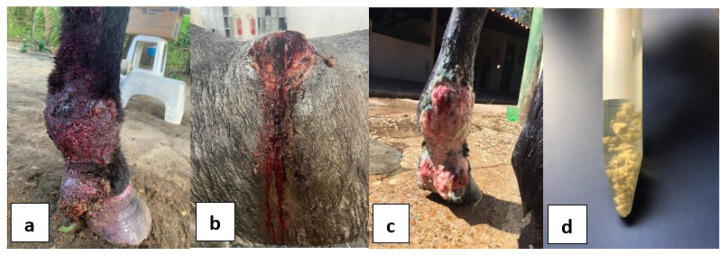
Equine study lesions (**a**–**c**) and kunkers in sterile antibiotic solution (**d**).

To verify the presence of *P. insidiosum*, all samples were subjected to microbiological culture with subsequent molecular detection of the grown colonies, and also molecular detection directly from the tissues. Small fragments of tissue samples were washed with sterile distilled water and antibiotics (Merck, Darmstadt, Germany). After that, the samples were inoculated on Sabouraud agar (SAB) (Merck, Darmstadt, Germany) and incubated at 37 °C for 5 to 7 days. The material was resuspended in SAB Dextrose (Merck, Darmstadt, Germany) broth with 150 rpm agitation at 37 °C for 5 days. Below you can see in Figure 1 images of the lesions in horses included in the study (a, b, c) and the leeches stored in sterile antibiotic solution (d).

DNA extraction was performed from animal tissue samples and colonies isolated on Sabouraud agar (SAB). Samples were washed with distilled water, crushed in a sterile mortar, and resuspended in lysis buffer (100 mM Tris-Cl, 50 mM EDTA, 2% SDS, 1% 2-mercapto ethanol), 10% CTAB (hexadecyltrimethylammonium bromide), and 5 N NaCl (Invitrogen, Waltham, MA, USA). DNA was extracted using the NucleoSpin^®^ Tissue kit (Macherey-Nagel, Düren, Germany) and eluted in 50 µL of Tris-HCl EDTA buffer (Invitrogen, Waltham, MA, USA) before storage at −20 °C. DNA concentration and quality were assessed through amplification of the endogenous equine GAPDH gene and spectrophotometric analysis at 260 nm using a NanoVue spectrophotometer (GE Healthcare, Chicago, IL, USA).

All positive samples underwent nested-PCR targeting the 18S ribosomal RNA gene (18S rDNA) using ITS1 forward (5′ TCC GTA GGT GAA CCT GCG G 3′) and ITS2p reverse (5′ TCC GCT TAT TGA TGC 3′) primers for the first round, and Pi1 forward (5′ TTC GTC GAA GCG GAC TGC T 3′) and Pi2 reverse (5′ GCC GTA CAA CCC GAG AGT CAT A 3′) primers for the second round [15]. The first amplification was conducted in 25 µL reactions containing 10 ng of genomic DNA, 1 µL of each primer (10 pmol/µL), 7.5 µL of ultrapure water (Merck, Darmstadt, Germany), and 12.5 µL of GoTaq^®^ Green Master Mix (Promega, Madison, WI, USA). Thermocycling conditions were: 95 °C for 3 min (initial denaturation), followed by 32 cycles of 95 °C for 30 s (denaturation), 57 °C for 30 s (annealing), 72 °C for 30 s (extension), and a final extension at 72 °C for 10 min. The second amplification used the same conditions but replaced the DNA template with 10 ng of the first-round product and adjusted the annealing temperature to 65 °C for 28 cycles. DNA obtained from *P. insidiosum* culture was used as a positive control.

Amplified products were visualized on a 1.5% agarose gel (Merck, Darmstadt, Germany) containing 1.0 µL/mL of Nancy-520 (Sigma-Aldrich, St. Louis, MO, USA). A 5 µL aliquot of the PCR product and a 100 bp molecular weight marker (Invitrogen, Waltham, MA, USA) were run in 1X TBE buffer (Merck, Darmstadt, Germany) at 100 V. Visualization was performed under UV light using Image Lab software v.6.1 (Bio-Rad, Hercules, CA, USA).

## 3. Results

There was microbiological growth only in Sample 2, from the region of Presidente Dutra, Maranhão. The presence of *P. Insidiosum* was confirmed by molecular diagnosis of the isolated culture. After being submitted to nested-PCR for detection of the agent, a 105 bp fragment.

The remaining kunkers samples received could not be cultured and conventional diagnosis was not possible. There was only bacterial growth resulting from secondary contamination, even after washing with antibiotic solution. This result was probably due to the delay between collection and processing of the clinical material in the laboratory.

There was amplification in four of the five animal samples (4/5), with the generation of 105 bp fragment amplicons in the second step of the nested-PCR. Sample 4 was not successful in detecting the agent, suggesting that the infectious cause of the lesion is another microorganism. These results can be seen in Table 2.

## 4. Discussion

Detection of *P. insidiosum* in four suspected samples, compared to microbial growth in only one sample, suggests that nested-PCR may offer greater sensitivity than conventional culture for diagnostic purposes, although this finding is based on a limited number of samples. Furthermore, the morphological identification of the pathogen is laborious and requires specialized knowledge. For this type of identification, it is necessary to induce the production of sexual structures in vitro, which is difficult [16]. These results provide the first molecular evidence of the agent in horses from Northeastern Brazil, where no previous studies using molecular detection have been reported.

Equine pythiosis remains an important challenge in tropical and subtropical regions, particularly in flood-prone environments. Although Brazil reports the highest prevalence worldwide, most molecular and serological studies have focused on the South and Central-West regions, leaving knowledge gaps in the Northeast. The present study contributes to filling this gap by providing molecular confirmation of *P. insidiosum* in horses from this underexplored area, reinforcing the importance of incorporating DNA-based tools into routine diagnosis.

In Thailand, pythiosis has been extensively documented in humans and animals, with high prevalence and diverse clinical manifestations confirmed by molecular methods [17,18]. Reports include, in addition to numerous human cases, the first confirmed case of nasal pythiosis in a horse, identified by molecular techniques [19]. In India, although most publications are focused on human infections, recent reviews highlight the growing relevance of pythiosis as a diagnostic challenge, particularly in cutaneous forms, emphasizing the role of molecular assays in differentiating it from fungal infections [20]. In the United States, the disease has been reported in both humans and horses, occurring more frequently in endemic areas such as Louisiana and Florida, and recent studies reinforce its wide geographic distribution and the need for molecular confirmation in suspected cases [21]. These findings reinforce that, regardless of geographic region, molecular detection is essential to confirm cases of pythiosis and to differentiate it from other clinically similar conditions.

In Egypt, Tartor et al. [22] described detailed clinicopathological findings, molecular confirmation, and genotyping of *P. insidiosum*, reinforcing the relevance of DNA-based methods for accurate pathogen identification. In Latin America, earlier reports such as those by Mendoza and Alfaro [23] in Costa Rica and Salas et al. [24] in Venezuela documented multiple equine cases, highlighting the endemicity of the disease in tropical regions and the predominance of cutaneous lesions.

Chaffin et al. [25] and Reis et al. [26] described severe and disseminated cases of equine pythiosis, demonstrating the potential of the disease to extend beyond the cutaneous form, especially when diagnosis is delayed. These reports illustrate the aggressive and potentially fatal nature of *P. insidiosum* infection in horses. In comparison, the present study, restricted to cutaneous lesions, corroborates that this is the most common form of presentation, but also reinforces the importance of early molecular confirmation to prevent progression to more severe conditions.

In Brazil, Paz et al. [27] reported an outbreak of equine pythiosis in the Southeastern region, in which environmental isolation and phylogenetic analysis of *P. insidiosum* were performed. This work is particularly relevant because it not only confirmed the pathogen in clinical samples but also demonstrated its presence in aquatic environments, reinforcing the ecological basis of transmission.

The correct and rapid diagnosis is fundamental for the recovery of equines suspected of having the disease. However, classical diagnostic techniques, such as microbiological culture and histopathology, have limitations; they usually take days to weeks to deliver results. Among the methods available for infectious disease diagnosis, molecular methods are undoubtedly the best performing in terms of sensitivity, precision and speed [28].

The microbiological growth of *P. insidiosum* was obtained in only one of the samples, highlighting the low sensitivity of this method for the diagnosis of equine pythiosis. This limitation may be attributed to the characteristics of the pathogen, which has specific growth requirements and is easily outcompeted by fast-growing environmental contaminants, in addition to the transport time to diagnostic centers, since the collection sites were located far away [29,30]. These factors make microbiological isolation not recommended as a routine technique [31].

The differential diagnosis of equine pythiosis involves some diseases of infectious origin, such as habronemosis, equine sarcoid and papillomatosis [12,13,32]. In the Northeast, the clinical and histopathological diagnosis, and eventually the microbiological one, are usually the only ones used for the confirmation of pythiosis. However, it is important to emphasize that studies of prevalence of pythiosis and other differentials in the Northeastern region of the country are not many in order to ensure that pythiosis is the main suspect, as well as the wrong diagnosis can result in ineffective treatment for the equine or even euthanasia due to the non-recovery of the animal [33].

Pythiosis is an eminent public health problem. Studies in Thailand have shown the occurrence of this disease in agricultural workers living in marshy areas, causing subcutaneous lesions, chronic inflammation of blood vessels, keratitis, aneurysms, and gangrene [34]. What may account to some extent for the higher occurrence of human pythiasis in Thailand is the higher presence of individuals with thalassemia, a predisposing factor for the development of *P. insidiosum* lesions [35]. Like fungal diseases, oomycotic diseases are often neglected by health professionals, and although some papers highlight the problem of neglected infections in Brazil and worldwide, they curiously do not mention or include pythiosis in this group [36,37,38].

The cases described in this study occurred in municipalities with very distinct environmental characteristics in Northeastern Brazil. While Mossoró (Rio Grande do Norte) is located in a semi-arid area but includes floodplains connected to the Apodi River, Paulista (Pernambuco) and João Pessoa (Paraíba) have a humid tropical climate, high rainfall, and coastal ecosystems with rivers, mangroves, and flooded areas. Palmeira dos Índios (Alagoas), located in the Agreste region, combines hilly relief, mountain ranges, and reservoirs that favor the formation of humid environments during the rainy season, although the analyzed case tested negative for *P. insidiosum*. In Presidente Dutra (Maranhão), in turn, the hot tropical climate with an intense rainy season also creates potentially favorable environmental conditions for the agent [39,40].

Despite regional differences, a common feature observed is the presence of water bodies or flooded environments, suggesting that regardless of the predominant climatic regime, water availability is a determining factor for the occurrence of equine pythiosis. These findings, although derived from a limited case series and from passive attendance at university hospitals, contribute to expanding knowledge on the distribution of the disease in Northeastern Brazil and highlight the importance of future studies to more systematically assess the relationship between environmental factors and the presence of the pathogen.

In Northeastern Brazil, the number of reported cases of equine pythiosis is limited, mainly due to the difficulty of accessing suspected animals in remote rural areas and the low demand for veterinary care by horse owners, who usually raise the animals for family work and with little professional assistance. During the study period, the cases described here were the only ones referred to the partner universities in the region. Despite the small number of samples, they represent occurrences in five different states, providing an initial but relevant overview of the disease in Northeastern Brazil. More advanced studies are needed from these agents, such as complete genome sequencing, in order to verify the genetic variability of the agent circulating in Brazil. Monitoring the occurrence of cases along with genomic studies for this species in Brazil is still lacking; however, analyses such as these are essential to establish the spatial distribution and verify if there is intraspecific diversity of *P. insidiosum* in Brazil as occurs in Thailand.

The small number of samples reflects the difficulty of accessing suspected cases in Northeastern Brazil, where outbreaks are located in areas far from diagnostic centers, limiting the generalization of the results. These were the only specimens submitted for analysis during the study period, highlighting both the logistical constraints and the low availability of clinical samples in the region.

It would be important to perform complementary analyses (such as broad-range PCR or sequencing) in the sample that tested negative for *P. insidiosum*. This issue is not restricted only to sample 4, but also to other suspected cases from the region that tested negative in both culture and PCR. Future studies should consider investigating alternative agents involved in granulomatous lesions in horses from Northeastern Brazil, which would allow a more comprehensive characterization of these clinical manifestations.

There is an urgent need for continuing education programs for breeders and rural producers [41,42]. This case report reinforces the importance of molecular diagnostic techniques for the confirmation of equine pythiosis. Thus, we support the need for further studies based on this one, in order to notify all Brazilian regions affected by pythiosis and investigate the genetic variability of the circulating agent in Brazil. The monitoring of the occurrence of cases along with studies for this species at a national level is still lacking; however, analyses such as these are essential to establish the spatial distribution of *P. insidiosum* and thus notify risk areas for affected animals in Brazil.

## 5. Conclusions

The presence of *P. insidiosum* was detected for the first time in four horse samples from northeastern Brazil by nested PCR directly from the tissues and kunkers of the lesions. Compared to microbial growth, the use of nested-PCR suggests greater sensitivity than conventional culture for diagnostic purposes, although this finding is based on a limited number of samples. This molecular tool represents a promising and potentially more economically attractive diagnostic option for breeders and veterinarians in the diagnosis of pythiosis-like lesions in horses from Northeastern Brazil.

## Figures and Tables

**Table 1:** Data regarding clinical samples from horses with lesions suggestive of pythiosis from five states in Northeastern Brazil.

Animal	Breed	Age	Sex	Source	Lesion Location
1	Mixed-breed	2 years	Male	Mossoró, Rio Grande do Norte	Abdominal Region
2	Mixed-breed	2 years	Female	Presidente Dutra, Maranhão	Left thoracic limb
3	Mixed-breed	3 years	Male	Paulista, Pernambuco	Abdominal Region
4	Mixed-breed	3 years	Male	Palmeira dos Índios, Alagoas	Left pelvic limb
5	Mixed-breed	3 years	Female	João Pessoa, Paraíba	Dorsal region

**Table 2:** Clinical samples from horses in Northeastern Brazil with granulomatous lesions and their respective results.

Animal	Material	Culture	PCR Endogenous Gene	Nested-PCR *P. insidiusum*
1	tissue	−	+	+
2	tissue	+	+	+
3	tissue and kunkers	−	+	+
4	tissue and kunkers	−	+	−
5	tissue	−	+	+

(+) positive result; (−) negative result.

## Data Availability

The data presented in this study are available on request from the corresponding author.
